# Enhancing biodesulfurization by engineering a synthetic dibenzothiophene mineralization pathway

**DOI:** 10.3389/fmicb.2022.987084

**Published:** 2022-10-05

**Authors:** Igor Martínez, Magdy El-Said Mohamed, José Luis García, Eduardo Díaz

**Affiliations:** ^1^Department of Microbial and Plant Biotechnology, Centro de Investigaciones Biológicas Margarita Salas-Consejo Superior de Investigaciones Científicas, Madrid, Spain; ^2^Research and Development Center, Saudi Aramco, Dhahran, Saudi Arabia

**Keywords:** *Pseudomonas azelaica*, dibenzothiophene, metabolic engineering, biodesulfurization, 4S pathway

## Abstract

A synthetic dibenzothiophene (DBT) mineralization pathway has been engineered in recombinant cells of *Pseudomonas azelaica* Aramco J strain for its use in biodesulfurization of thiophenic compounds and crude oil. This functional pathway consists of a combination of a recombinant 4S pathway responsible for the conversion of DBT into 2-hydroxybiphenyl (2HBP) and a 2HBP mineralization pathway that is naturally present in the parental *P. azelaica* Aramco J strain. This novel approach allows overcoming one of the major bottlenecks of the biodesulfurization process, i.e., the feedback inhibitory effect of 2HBP on the 4S pathway enzymes. Resting cells-based biodesulfurization assays using DBT as a sulfur source showed that the 2HBP generated from the 4S pathway is subsequently metabolized by the cell, yielding an increase of 100% in DBT removal with respect to previously optimized *Pseudomonas putida* biodesulfurizing strains. Moreover, the recombinant *P. azelaica* Aramco J strain was able to use DBT as a carbon source, representing the best characterized biocatalyst harboring a DBT mineralization pathway and constituting a suitable candidate to develop future bioremediation/bioconversion strategies for oil-contaminated sites.

## Introduction

**Figure 1:**
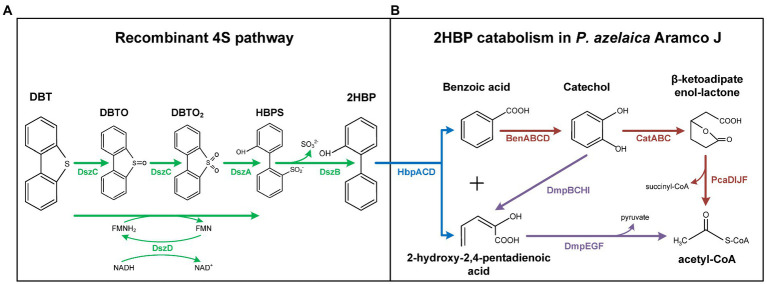
Synthetic DBT degradation pathway in recombinant *Pseudomonas azelaica* AJdsz strain. The synthetic pathway was constructed by coupling a recombinant 4S pathway **(A)** with a 2HBP degradation pathway naturally present in *P. azelaica* Aramco J **(B)**. Green: dsz pathway; blue: hbp pathway; brown: ben-cat-pca pathways; purple: dmp pathway. DBT, dibenzothiophene; DBTO, dibenzothiophene sulfoxide; DBTO_2_ dibenzothiophene sulfone; HBPS, 2-hydroxybiphenyl-2-sulfinate; 2HBP, 2-hydroxybiphenyl.

Over the last decades, biodesulfurization (BDS) has become an attractive approach that aims to complement the traditional hydrodesulfurization treatment of crude oils and refined products to specifically remove sulfur from *S*-heterocyclic compounds that are recalcitrant to the chemical treatments (Martínez et al., 2017). Dibenzothiophene (DBT) and its alkylated derivatives are the prototypical compounds targeted in BDS (Monticello, 2000; Soleimani et al., 2007). The most studied pathway to remove sulfur from DBT is the 4S pathway (Figure 1A). The 4S route is a non-destructive aerobic pathway that converts DBT into 2-hydroxybiphenyl (2HBP) through two oxidation steps, catalyzed by the DszC and DszA monooxygenase enzymes, followed by a hydrolytic step, mediated by the DszB desulfinase, which are usually plasmid encoded. The FMNH_2_ required for the oxidation steps is supplied by a NADH-FMN oxidoreductase (DszD; Figure 1A), which is usually chromosomally encoded (Gupta et al., 2005; Mohebali and Ball, 2016; Kilbane, 2017; Parveen et al., 2020; Hirschler et al., 2021; Martín-Cabello et al., 2022).

One of the major bottlenecks of the biodesulfurization of DBT through the 4S route is the inhibitory effect of the final product, 2HBP, on the initial Dsz enzymes (Xu et al., 2006; Chen et al., 2008; Akhtar et al., 2009; Abin-Fuentes et al., 2013; Kilbane, 2017; Li et al., 2019a). To deal with this limitation, different approaches have been applied over the years. Optimization of the oil/aqueous phase ratio is an option to weaken the feedback inhibition effects (Feng et al., 2016). Compartmentalization of the individual reactions of the 4S pathway into different hosts allows for their individual optimization to overcome 2HBP feedback inhibition (Martínez et al., 2016). The use of 2HBP-reasonably resistant bacteria harboring the 4S pathway can handle the inhibitory effects of 2HBP in cell growth (Akhtar et al., 2018). Directed evolution combined with a high-throughput screening method, combinatorial mutagenesis and enzyme overexpression generated a DszC mutant enzyme that was less sensitive to 2HBP feedback inhibition than the parental enzyme (Li et al., 2019a). Other alternative is the use of an extended 4S pathways where the final product, e.g., 2-methoxybiphenyl, has less inhibitory effects than 2HBP (Li et al., 2003; Chen et al., 2009; Bordoloi et al., 2014, 2016).

In this work, we propose a new approach to overcome the 2HBP-mediated inhibition of biodesulfurization through the expression of the 4S pathway in a 2HBP-degrading bacterium, *Pseudomonas azelaica* strain Aramco J (Mohamed et al., 2015), that is highly similar (99% nucleotide identity) to the well-studied *Pseudomonas nitroreducens* strain HBP-1 that can naturally export and catabolize 2HBP using the tripartite complex pump MexAB-OprM and HbpCAD enzymes, respectively (Jaspers et al., 2001; Czechowska et al., 2013; Carraro et al., 2020). The engineered *P. azelaica* strain is the first rationally-designed biocatalyst that couples the 4S pathway to the subsequent degradation of 2HBP, yielding a synthetic pathway that significantly increases DBT biodesulfurization yield, and allows the use of DBT as a carbon source.

## Materials and methods

### Chemicals and culture media

Suppliers of the different chemicals used were as follows: 2HBP was from Fluka; DBT, HEPES, glutamic acid, glucose, Tween 80, gentamicin, kanamycin and isopropyl β-D-thiogalactopyraniside (IPTG) were from Sigma-Aldrich. All the chemicals used to prepare the different media were from Sigma-Aldrich. Deionized water (resistivity 18.5 MΩ cm) was used to prepare all of the media.

Lysogeny broth (LB) medium containing 1% tryptone, 0.5% yeast extract and 1% NaCl was used for the pre-cultures (Miller, 1972). Sulfur-free basal salts medium (BSM) employed for the growth experiments using DBT (0.1 mM) as a sulfur source had the following composition (Martín et al., 2004): NaH_2_PO_4_·H_2_O 4 g/l; K_2_HPO_4_·3H_2_O 4 g/l; NH_4_Cl 2 g/l; MgCl_2_·6H_2_O 0.0245 g/l; CaCl_2_·2H_2_O 0.001 g/l; and FeCl_3_·6H_2_O 0.001 g/l. M63 medium was used for the growth experiments using DBT (2.5 mM) as a carbon source and had the following composition (Miller, 1972): KH_2_PO_4_ 13.6 g/l; (NH_4_)SO_4_ 2 g/l; FeSO_4_·7H_2_O 0.5 mg/l, adjusted to pH 7.0 using KOH, with trace solutions (MnCl_2_·4H_2_O 1.98 mg/l; CoSO_4_·7H_2_O 2.81 mg/l; CaCl_2_·2H_2_O 1.47 mg/l; CuCl_2_·2H_2_O 0.17 mg/l and ZnSO_4_·7H_2_O 0.29 mg/l). Due to the low solubility of DBT, this medium was sonicated for 30 min in a sonication bath to maximize the particle dispersion and obtain a homogeneous distribution without visually aggregated particles of DBT.

Resting cells assays were conducted in 50 mM HEPES buffer at pH 8.0 supplemented with Tween 80 (0.1%) and sonicated for 30 min in a sonication bath after DBT addition.

### Bacterial strains, plasmids and growth conditions

Bacterial strains, plasmids and primers used in this work are listed in Table 1. *Escherichia coli* strains were grown in LB medium at 37°C and 200 rpm. *P. azelaica* and *Pseudomonas putida* strains were grown at 30°C in an orbital shaker at 200 rpm in BSM or M63 media. When appropriate, antibiotics were added at the following concentrations: ampicillin (100 μg mL^−1^), kanamycin (50 μg mL^−1^), gentamicin (10 μg mL^−1^).

**Table 1:** Bacterial strains, plasmids and primers used in this work.

Strain	Relevant genotype–phenotype	Reference/source
*E. coli* DH10B	F^−^, *mcrA,* Δ(*mrrhsdRMS-mcrBC*), Ф80d*lacZ*ΔM15, Δ*lacX74*, *deoR*, *recA1, araD139,* Δ(*ara-leu*)7697, *galU, galK,* λ^−^, *rpsL, endA1, nupG*	Life technologies
*P. azelaica* AJ	wild type strain, 2HBP^+^	Mohamed et al. (2015)
*P. azelaica* AJdsz	*P. azelaica* AJ strain harboring plasmid pIZdszB1A1C1-D1, Gm^r^	This study
*P. azelaica AJdhbpA*	*P. azelaica* AJ mutant strain with a disruption on the *hbaA* gene, Km^r^, 2HBP^−^	This study
*P. azelaica* AJd*hbpA*dsz	*P. azelaica* AJd*hbpA* strain harboring plasmid pIZdszB1A1C1-D1, Km^r^, Gm^r^	This study
*P. putida* KT2440 (pIZdszB1A1C1-D1)	*P. putida* KT2440 strain harboring plasmid pIZdszB1A1C1-D1, Gm^r^	Martínez et al. (2016)
Plasmid	Description	Reference/source
pGEM-T Easy	Ap^r^, *ori*ColE1, *lacZα*, used for cloning PCR products	Promega
pGEM-hbpA	Ap^r^, pGEM-T Easy containing a 1,100 bp *Eco*RI *hbpA* internal fragment	This study
pK18*mob*	Km^r^, *ori*ColE1, Mob^+^, *lacZα*, used for directed insertional disruption	Schäfer et al. (1994)
pK18*mobhbpA*	Km^r^, pK18*mob* containing a 1,100 bp *Eco*RI *hbpA* internal fragment	This study
pIZdszB1A1C1-D1	Gm^r^ pIZ1016 derivative expressing the synthetic *dszB1A1C1-D1* cassette	Martínez et al. (2016)
Primers	Sequence	Reference/source
hbpA-Fw	AATTATGAATTCGAGCACGAGCTGGCAAGCCCATC	This study
hbpA-Rv	AATTATGAATTCCTGGCCGTGTACTGGCCTGATAG	This study
Ext-hbpA-Fw	GCGCTATGTCGGCGACTTTGTTG	This study
Ext-hbpA-Rv	CCAAGGATGCTCTTCACTGCCACG	This study

### Growing cells experiments

All experiments started with overnight pre-cultures in LB medium containing the appropriate antibiotics. For the growth experiments using DBT as a sulfur source, *P. azelaica* pre-cultures were washed twice with saline solution (0.9% NaCl, w/v) and used to inoculate (initial OD_600_ of 0.1) 50 ml of sulfur-free BSM medium supplemented with glucose (2 g/l), glycerol (2.45 g/l), 0.1 mM DBT and 1 mM IPTG in 250 ml Erlenmeyer flasks.

For the growth experiments using DBT as a carbon source, *P. azelaica* pre-cultures were washed twice with saline solution and used to inoculate (initial OD_600_ of 0.1) 50 ml of pre-sonicated M63 medium supplemented with 6.8 mM glutamic acid, Tween 80 (0.1%) and 2.5 mM DBT or 2.5 mM 2HBP in 250 ml Erlenmeyer flasks.

Optical density at 600 nm (OD_600_) was measured using a Shimadzu UV–vis spectrophotometer (model UV mini 1,240). Due to the high turbidity of the M63 medium when 2.5 mM DBT was added, growth under these conditions was determined as colony forming units (CFUs) by plating culture samples in LB solid medium.

### Resting cells assays

For the resting cells assays, *P. azelaica* and *P. putida* strains were grown overnight in LB medium containing the appropriate antibiotics. Then, cultures were washed twice with saline solution and used to inoculate 50 ml of M63 medium supplemented with glutamic acid (20 g/l), 1 mM IPTG and the appropriate antibiotic. After 24 h, cultures were washed twice in saline solution and resuspended in HEPES buffer supplemented with Tween 80 (0.1%) and 0.5 mM DBT at an OD_600_ of 2.0 (about 1 g/l cell dry weight) to start the resting cells assays.

Samples were collected periodically and mixed with an equal volume of acetonitrile, then centrifuged at 14,000 ×*g* for 10 min and filtered through 0.22 μm polyethersulfone filters (Minisart high flow, Sartorius) for the subsequent HPLC analysis.

### Molecular biology techniques

Standard molecular biology techniques were performed as previously described (Sambrook and Russell, 2001). Plasmid DNA was prepared with a High Pure plasmid isolation kit (Roche Applied Science). DNA fragments were purified with Gene-Clean Turbo (Q-BIOgene). Oligonucleotides were supplied by Sigma. All cloned inserts and DNA fragments were confirmed by DNA sequencing through an ABI Prism 377 automated DNA sequencer (Applied Biosystems Inc.). Transformation of bacterial cells was carried out by electroporation (Gene Pulser; BioRad) (Sambrook and Russell, 2001).

### Construction of *Pseudomonas azelaica* AJd*hbpA* strain

For insertional disruption of the *hbpA* gene through single homologous recombination, a 1.1 kb internal region of *hbpA* gene was PCR-amplified using primers hbpA-Fw and hbpA-Rv and cloned into *Eco*RI site of vector pGEM-T Easy giving rise to plasmid pGEM-hbpA (Table 1). The pGEM-hbpA plasmid was digested with *Eco*RI, and the 1.1 kb fragment was then subcloned into the pK18*mob* suicide vector giving rise to the pK18*mobhbpA* plasmid. The pK18*mobhbpA* plasmid was purified and transformed by electroporation into *P. azelaica* AJ. The recombinant strain, harboring the disrupted *hbpA* gene by insertion of the pK18*mobhbpA* suicide plasmid, was isolated on kanamycin-containing LB agar plates. The mutant strain was finally analyzed by PCR to confirm the disruption of *hbpA* gene.

### Analytical methods

HPLC (Agilent 1100 Series) was employed to analyze the concentration of DBT and 2HBP using a C18 column (Teknokroma C18 150×4.6 mm, 5 μm particles) at 1 ml/min flow rate. The mobile phase was a mixture acetonitrile/water (55:45). Peaks were monitored at different wave lengths (234 nm for DBT and 206 nm for 2HBP). Calibrations were performed using highly purified standards of each compound.

## Results and discussion

### Engineering a heterologous 4S biodesulfurization pathway in recombinant *Pseudomonas azelaica* Aramco J strain

*Pseudomonas azelaica* strain Aramco J (*P. azelaica* AJ) was isolated from an oil-contaminated soil sample from Abu Ali Island (Saudi Arabia) and is naturally adapted to degrade 2HBP as well as other hydroxybiphenyls (Mohamed et al., 2015). Growth experiments revealed that *P. azelaica* AJ was able to grow in the presence of up to 15 g/l 2HBP. Degradation of 2HBP is carried out by the hbp pathway that generates benzoate and 2-hydroxy-pentadienoate (Figure 1B; Jaspers et al., 2001). The *hbpACD* genes are located within an integrative-conjugative element (ICEhbp) in the genome of *P. azelaica* AJ (Mohamed et al., 2015). Next to the *hbp* genes in the ICEhbp element are located the *dmp* genes encoding a catechol *meta*-cleavage pathway also involved in the metabolism of 2-hydroxy-pentadienoate (Figure 1B). The degradation of benzoate involves its initial conversion to catechol by the *benABCD* gene products (Figure 1B). Catechol can be then catabolized through the *dmp meta-*cleavage pathway or *via* the *cat ortho*-cleavage pathway (Figure 1B). Two different *ben-cat* gene clusters are located outside the ICEhbp element, and they are mapped at different positions of the Aramco J genome (Mohamed et al., 2015). The final product of the *cat* pathway, i.e., β-ketoadipate enol-lactone, is finally converted to Krebs-cycle intermediates through the β-ketoadipate pathway (Figure 1B) which is encoded by two *pca* gene clusters, one located within ICEhbp element and the other associated to one of the *ben-cat* clusters (Mohamed et al., 2015).

In a previous work, we have constructed a synthetic 4S pathway (dszB1A1C1-D1 cassette) that enhanced the bioconversion of DBT to 2HBP when expressed in *P. putida* KT2440 (Martínez et al., 2016). Plasmid pIZdszB1A1C1-D1 is a pIZ1016-derived plasmid that harbors the synthetic *dszB1, dszA1* and *dszC1* genes under control of the IPTG-dependent *P*_*tac*_/*lac*I^q^ regulatory system, and dszD1 gene under control of *P*_*tac*_/*lac*I^q^ regulatory system (Martínez et al., 2016). When plasmid pIZdszB1A1C1-D1 was transferred to *P. azelaica* AJ, the resulting strain *P. azelaica* AJ (pIZdszB1A1C1-D1), named *P. azelaica* AJdsz, was predicted to acquire the ability to transform DBT into 2HBP by the 4S pathway with the subsequent degradation of 2HBP by the endogenous metabolic network of the host strain (Figure 1). To confirm this assumption, we performed desulfurization experiments by growing cells in the presence of DBT.

**Figure 2:**
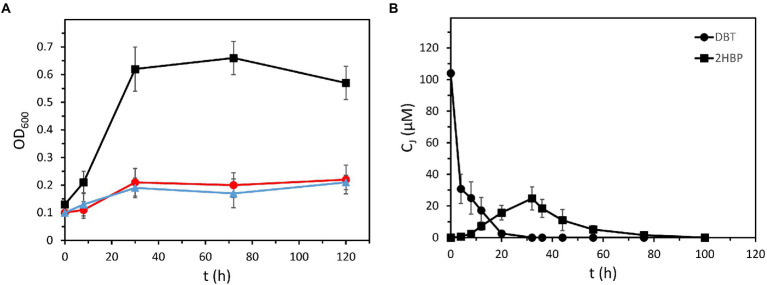
Growth of recombinant *P. azelaica* AJdsz using DBT as the only sulfur source. **(A)** Growth of recombinant *P. azelaica* AJdsz in BSM containing 100 μM of DBT as the only sulfur source, 0.2% (w/v) glucose as carbon source, and 1 mM IPTG to induce the *dsz* gene expression (black squares). Control experiments of *P. azelaica* AJdsz without DBT (blue triangles) and wild-type *P. azelaica* AJ strain with DBT (red circles) are shown. **(B)** Time course of the evolution of DBT (black circles) and 2HBP (black squares) during growth of recombinant *P. azelaica* AJdsz in DBT as only sulfur source. C_j_ indicates the concentration of the monitored compound. Values are the mean of three different experiments. Error bars indicate standard deviations.

In contrast to the wild-type *P. azelaica* AJ strain, the recombinant *P. azelaica* AJdsz was able to grow using 0.1 mM DBT as the sole sulfur source when IPTG was added to the culture medium (Figure 2A), hence demonstrating that the heterologous 4S pathway was successfully expressed in the recombinant *P. azelaica* AJdsz strain. Analyzes of the culture supernatants along the growth curve confirmed that all DBT was removed during the exponential growth phase in about 20 h (Figure 2B). Although some 2HBP accumulated in the supernatants during DBT consumption reaching a maximum of 23 μM at 24 h, it was subsequently degraded until its complete removal (Figure 2B). These results further confirm that *P. azelaica* AJdsz is able to use sulfur from DBT through the 4S pathway without accumulating 2HBP as final product, thus suggesting that 2HBP is efficiently metabolized by the action of the endogenous *hbp* genes avoiding the 2HBP-dependent feedback inhibition of Dsz enzymes. The initial accumulation of 2HBP in the culture medium is likely due to the fact that the initial biodegradation steps of DBT are expressed in a multicopy plasmid under the control of strong promoters, whereas the degradation pathway of 2HBP is expressed in a single copy in the chromosome. The fine tuning between both pathways to avoid the accumulation of intermediates, a property that is acquired during evolution, can be achieved in the future by changing gene copy number and the promoters. Moreover, it should be mentioned that a search in the genome of strain Aramco J revealed the presence in contig 7 of a *mexABoprM* cluster highly similar to that reported in *P. nitroreducens* HBP-1 strain and that encodes an efficient 2HBP efflux system that is crucial to secrete 2HBP and tolerate high 2HBP concentrations (Czechowska et al., 2013). Secretion of 2HBP via the MexAB-OprM efflux pump could also contribute to the action of the HbpCAD enzymes metabolizing 2HBP to prevent high 2HBP intracellular accumulation and thus, helping to reduce feedback inhibition of the Dsz enzymes. Interestingly, neither in strain HBP-1 nor in Aramco J strain the *mexABoprM* cluster is linked to the catabolic *hbpCAD* genes but rather it is located elsewhere on the chromosome (Carraro et al., 2020).

Nevertheless, to check whether growth on 2HBP could inhibit the Dsz enzymes in *P. azelaica* AJdsz, this recombinant strain and the wild-type *P. azelaica* AJ strain were grown in minimal medium with 2 mM 2HBP as carbon source and 0.1 mM DBT or 0.1 mM sulfate as sole sulfur sources (Table 2). As expected, *P. azelaica* AJ was able to grow using sulfate but it did not grow in the presence of DBT. In contrast, the strain AJdsz was able to grow under both conditions (Table 2), indicating that the *dsz* pathway expressed in *P. azelaica* AJdsz is functional even when growing the cells in the presence of high amounts of 2HBP.

**Table 2:** Growth of *P. azelaica* AJ strains in BSM with 2HBP as a carbon source and SO_4_ or DBT as a sulfur source.

Strain	SO_4_ (mM)	DBT (mM)	2HBP (mM)	OD_600_ (24 h)^*^	OD_600_ (48 h)^*^
*P. azelaica* AJdsz	-	0.1	2	0.22	0.44
*P. azelaica* AJdsz	0.1	-	2	0.42	0.49
*P. azelaica* AJdsz	-	-	2	0.14	0.12
*P. azelaica* AJ	-	0.1	2	0.12	0.12
*P. azelaica* AJ	0.1	-	2	0.41	0.52
*P. azelaica* AJ	-	-	2	0.14	0.16

* Values of one experiment are shown, and they were reproducible in three different experiments with standard deviations lower than 10%.

All these results taken together suggest that the efficient metabolism of 2HBP in *P. azelaica* AJdsz reduces the inhibitory effects of this 4S pathway intermediate, and encourage the use of this recombinant strain as a suitable biocatalyst for DBT desulfurization.

### Recombinant *Pseudomonas azelaica* AJdsz strain as a biocatalyst for DBT desulfurization

**Figure 3:**
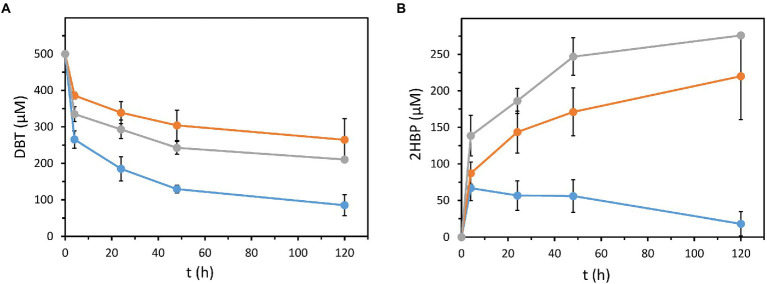
DBT degradation and 2HBP accumulation in resting cells assays. Strains tested were: *P. azelaica* AJdsz (blue circles), *P. azelaica* AJd*hbpA*dsz (gray triangles) and *P. putida* KT2440 harboring plasmid pIZdszB1A1C1-D1 (orange squares). **(A)** Evolution of DBT concentration in resting cells assays containing 500 μM of DBT as initial sulfur substrate. **(B)** Evolution of 2HBP concentration. Values are the mean of three different experiments. Error bars indicate standard deviations.

As suggested above, the expression of the 4S pathway in a 2HBP degrading bacterium, as *P. azelaica* AJdsz, reduces the 2HBP-dependent inhibition of Dsz enzymes by efficiently degrading this intermediate. To confirm this hypothesis, the desulfurizing capacity of *P. azelaica* AJdsz was tested using 500 μM DBT in resting cell assays. *P. azelaica* AJdsz was able to efficiently remove about 85% of the initial DBT after 120 min (Figure 3A) with almost no net accumulation of 2HBP (Figure 3B). This result contrasts with that obtained under the same conditions with our best desulfurizing strain so far, i.e., *P. putida* KT2440 (pIZdszB1A1C1-D1; Martínez et al., 2016), that was able to remove only 40% of the initial DBT (Figure 3A) and accumulated an equimolar amount of 2HBP (about 200 μM, Figure 3B). Thus, *P. azelaica* AJdsz strain behaved as a more efficient DBT biodesulfurizer than the previously engineered *P. putida* strain, in agreement with the expected lower 2HBP accumulation in the resting assays.

To confirm that the significant increase of DBT desulfurization in *P. azelaica* AJdsz strain was due to the metabolism of the released 2HBP, we engineered a mutant *P. azelaica* AJ strain lacking a functional HbpA enzyme responsible for the first step in the 2HBP catabolism. The mutant strain, *P. azelaica* AJd*hpbA*, was transformed with plasmid pIZdszB1A1C1-D1 and, as expected, the resulting strain *P. azelaica* AJd*hpbA*dsz was unable to grow using 2HBP as carbon source. The recombinant strain *P. azelaica* AJd*hpbA*dsz was able to desulfurize DBT *via* the 4S pathway, although it was only able to eliminate 50% of the initial DBT (Figure 3A) accumulating equimolar amounts of 2HBP in the culture medium (Figure 3B). This behavior was similar to that of *P. putida* KT2440 (pIZdszB1A1C1-D1), another strain unable to degrade 2HBP (Martínez et al., 2016), and contrasts with the higher desulfurization efficiency of the *P. azelaica* AJdsz strain able to metabolize 2HBP (Figure 3). Therefore, all these results taken together revealed that a bacterial host cell able to metabolize 2HBP and expressing the *dsz* genes, as *P. azelaica* AJdsz, constitutes a successful DBT biodesulfurizer strain that reaches one of the highest specific desulfurization activities (about 42 μmol_DBT_/g DCW/h) reported so far when using microbial resting cell cultures in aqueous phase (Martínez et al., 2016; Li et al., 2019a).

### Use of DBT as a carbon source by recombinant *Pseudomonas azelaica* AJdsz strain

**Figure 4:**
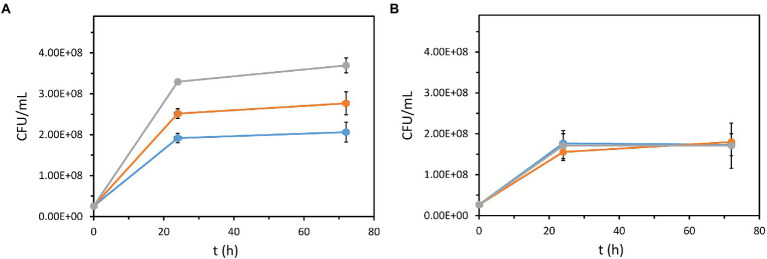
Growth of recombinant *P. azelaica* strains using DBT or 2HBP as a carbon source. *P. azelaica* AJdsz **(A)** and *P. azelaica* AJd*hbpA*dsz **(B)** were grown in M63 medium with 6.8 mM glutamic acid (basal carbon and energy source) in the absence (blue lines) or in the presence of 2.5 mM DBT (orange lines) or 2.5 mM 2HBP (gray lines). Due to the high turbidity of the medium, growth was determined as CFU/mL (1 gDCW/L = 5.2·10^8^ CFU/ml). Values are the mean of three different experiments. Error bars indicate standard deviations.

Another important property of the recombinant *P. azelaica* AJdsz strain developed in this work is that this strain has the enzymatic machinery required to use DBT as a carbon source (Figure 1). To test the mineralization of DBT in *P. azelaica* AJdsz strain, initial tests were carried out in minimal medium with DBT as the sole carbon and energy source, but no apparent growth was observed. Since the 4S pathway requires high amounts of NADH (Galán et al., 2000; Abin-Fuentes et al., 2013), it might be necessary the use of an additional carbon source to provide the energy required for the initial degradation of DBT. Thus, we checked the growth of *P. azelaica* AJdsz by counting the colony forming units (CFU/ml) in minimal medium with 2.5 mM DBT (30 mM total carbon) in the presence of 6.8 mM glutamic acid (34 mM total carbon) as additional carbon and energy source (Martín et al., 2004). The results obtained revealed that the addition of 2.5 mM DBT increased cell growth with respect to the control experiment without DBT (Figure 4A). Nevertheless, the CFU value was lower than that obtained using 2.5 mM 2HBP (30 mM total carbon) as carbon and energy source (Figure 4A). HPLC analysis of the culture supernatants at 72 h revealed that whereas all 2HBP was consumed, only about 0.7 mM DBT (8.4 mM total carbon) was removed without accumulating 2HBP. This result explains the higher CFU value observed when the cells grew in 2HBP. Interestingly, when we used the *P. azelaica* AJd*hbpA*dsz mutant strain unable to catabolize 2HBP we did not observe any increase of cell growth in DBT or 2HBP with respect to the control experiment without such carbon source (Figure 4B). It is possible that DBT degradation may deliver carbon atoms at quite different rate than glutamate and 2HBP due to the relative insolubility of the DBT substrate. Therefore, these results allow us to conclude that 2HBP generated from DBT desulfurization *via* the 4S pathway is finally mineralized in strain *P. azelaica* AJdsz, likely by using the catabolic scheme depicted in Figure 1.

Polyaromatic sulfur heterocycles can persist over the long term with low bioavailability under field conditions and they are harmful to their surroundings. DBT, in particular, has been confirmed to be carcinogenic, teratogenic, and mutagenic to humans and other species either by ingestion, touch, or inhalation even at trace concentrations (Kirimura et al., 2001; Xu et al., 2006; Li et al., 2012). Surprisingly, despite DBT has been extensively studied as a sulfur source in desulfurization processes, there are only a few works dealing with the use of DBT as a carbon source in bacteria, and there is a lack of knowledge on the metabolic pathways responsible for this catabolism. Attempts to study DBT as C source have been unsuccessful partly because of the very low solubility of DBT relative to C demand, and partly because expression of the main known desulfurization genes is repressed by the excess of sulfate generated in the 4S pathway. The production of benzoate from DBTO sulfoxide/sulfone and its further degradation has been proposed in *Brevibacterium* (Van Afferden et al., 1990) and *Arthrobacter* DBTS2 (Sato and Clark, 1996). The introduction of the *carABC* operon of *Pseudomonas* CA10 in the DBT desulfurizer *Rhodococcus* XP strain suggested that the carbazole-degrading enzymes can attack the 2HBP as it was possible to detect benzoate in the medium (Yu et al., 2006). Different isolates of the genus *Bacillus* (Buzanello et al., 2014) and *Cobetia* (Ibacache-Quiroga et al., 2013) were able to grow in a medium supplemented with DBT as carbon source. Using benzoate as carbon source, *Enterobacter* sp. strain NISOC-03 was able to use DBT and produce 2HBP during the exponential growth phase and subsequently degrade it in the stationary growth phase, but the metabolic pathway involved has not been elucidated (Papizadeh et al., 2017). Recently a multiple metabolic pathway, with the production of at least twenty-six different metabolites, was proposed for the degradation of DBT in *Pseudomonas* sp. LKY-5 (Li et al., 2019b). The synthetic pathway generated in *P. azelaica* AJdsz (Figure 1) constitutes the best characterized mechanism of DBT degradation reported so far, and it could be easily expanded to and tested in other 2HBP bacterial degraders.

## Conclusion

We have assembled the first synthetic pathway to completely metabolize DBT. The expression of the 4S pathway *dsz* genes in a 2HBP-degrading bacterium (*P. azelaica* Aramco J strain) was shown to mitigate a major bottleneck, i.e., the 2HBP-mediated inhibition of Dsz enzymes, hence significantly improving the yield of DBT biodesulfurization. Moreover, the *P. azelaica* AJdsz strain becomes also a suitable biocatalyst not only to completely degrade DBT for the bioremediation of polyaromatic sulfur heterocycles-contaminated sites and sediments, but also to transform it into value-added compounds taking advantage of the complex metabolism present in *P. azelaica* Aramco J. Future work should be performed to enhance DBT metabolism for real-world applications, e.g., evaluating the biodesulfurization process of crude oil in biphasic systems or the remediation of crude in contaminated sites.

## Data availability statement

The original contributions presented in the study are included in the article/supplementary material, further inquiries can be directed to the corresponding author.

## Author contributions

IM performed the experiments. IM, MM, JG, and ED designed the experiments, contributed to the discussion, and interpretation of the data. IM, JG, and ED wrote the article. All authors contributed to the article and approved the submitted version.

## Funding

This work was supported by Saudi Aramco, and by grants BIO2016-79736-R, RTI2018-095584-B-C44, PID2019-110612RB-I00, and PCI2019-111833-2 from the Ministry of Science and Innovation of Spain; by grant CSIC 2019 20E005, and by European Union H2020 Grant 101000733.

## Conflict of interest

Authors declare that the research was conducted in the absence of any commercial or financial relationships that could be construed as a potential conflict of interest.

## Publisher’s note

All claims expressed in this article are solely those of the authors and do not necessarily represent those of their affiliated organizations, or those of the publisher, the editors and the reviewers. Any product that may be evaluated in this article, or claim that may be made by its manufacturer, is not guaranteed or endorsed by the publisher.
